# The Medical Society

**Published:** 1896-12-26

**Authors:** 


### Medical Progress and Hospital Clinics.

[*The Editor will be glad to receive offers of co-operation and contributions from members of the profession. All letter should be addressed to* The Editor, at the Office, 28 & 29, Southampton Street, Strand, London, W.C.]

### THE MEDICAL SOCIETY.

At the last meeting of the Medical Society, Professor Mayo Robson brought forward an interesting series of cases of appendicitis associated with general peritonitis, together with other cases on which he had operated during the year. Like many other practical surgeons constantly engaged in operative work, he tended in his classification to eschew pathology, and divided his cases in accordance with the treatment required rather than with the cause which had set up the mischief, for, as he said, the most important points to settle in any case of appendicitis were: Ought an operation to be done? and, if so, when? After considerable experience, his conviction was that the early operation, undertaken as soon as appendicitis was diagnosed, would lead to a far greater percentage of recoveries than the method of individualising which we still adopted in England. This conviction, however, arose from his knowledge that the removal of an appendix before suppuration, perforation, or gangrene had occurred, or before firm adhesions had formed, was practically unattended with risk, as was shown by the experience of Mr. Treves, himself, and many other surgeons. The operation done under such circumstances could be accomplished through a small incision, with separation rather than division of muscular fibres, whereas if resolution were waited for serious difficulties were not uncommonly met with, and it might become necessary to operate under very unfavourable conditions. Although, however, before operation the clinical division was into cases which should be operated on, and those which might be for the time left alone, after operation it was easy to divide them into catarrhal and suppurative—the latter being either without perforation, or with perforation, or associated with gangrene, although without perforation. Catarrhal appendicitis furnished the majority of recurrent cases, the variety which might, and usually did, recover without surgical treatment. But although the first and second attacks in such cases might be catarrhal, there was no guarantee that subsequent attacks would be of the same character. At any time the very next attack might turn out to be one of suppurative appendicitis, demanding immediate operation. Suppurative appendicitis might be either acute or chronic, but whatever course it assumed it demanded operation, which should be advised at the earliest possible moment, as soon as ever a probable diagnosis was arrived at. When appendicitis came on acutely with a rapid pulse and tenderness over the appendix, but without the presence of a tumour, the case should be looked on as one demanding immediate operation; while a more gradual onset with a quiet pulse (not over 100) and the early formation of a tumour were signs that delay might be safe. But, in the course of an appendicitis, whatever its apparent character, the occurrence of a rigor with rise of pulse and temperature should be looked on as indicating septic absorption and pointing to the necessity for operation. The pulse rather than the temperature was the true guide to treatment. Relief or cessation of pain, with a marked rise in the pulse rate, was an indication for immediate operation, as it frequently meant gangrene of the appendix; while distension of the abdomen, with vomiting and rapid pulse, were signs that admitted of no delay, as they were indicative of extending or general peritonitis. The effect of opium in disguising the symptoms must be remembered, and if it had been given it might be necessary to delay giving a decided opinion until it had been withheld for a few hours; when, if the pulse increased in frequency, or there was anxiety of countenance, operation would be required. In regard to the time when the operation should be done, he thought that in the subacute or catarrhal form the quiescent period was the safest, so that any time after the subsidence of the more acute symptoms might be selected. But an appendix, after it had once been inflamed, was very apt to give repeated and often fatal trouble in the future; and, although English practice was not so radical, he thought it might be sound advice to advocate the removal of the appendix after the first attack. In any case, however, he did most strongly maintain that after a second attack operation should be decidedly urged, as repeated seizures would certainly occur, and although catarrhal at first, they might at any time become suppurative. In regard to the extent of the operation, his feeling was that with few exceptions it should be made complete by removing the origin of the trouble; but in some cases it might not be possible to do this, either from the patient being too ill to bear the search or from it not being possible to find the appendix without greater separation of adhesions than might seem prudent. The only cases in which this question was likely to arise were those in which the suppuration was local, and where there was fear of opening the general peritoneal cavity. In the recurrent form there could be no doubt about it, as the removal of the appendix was the very object of the operation, and where there was general peritonitis the perforated or gangrenous appendix should be taken away. As to results, he thought there ought to be no mortality in operations undertaken in the quiescent period, and a very slight one in acute suppurative case where the area of suppuration was limited; the matter was different, however, where the appendicitis was accompanied with general suppurative peritonitis. In such cases he believed in very free irrigation with plain boiled water, or with weak boracic lotion, and in very free drainage by several large rubber tubes. It must be understood, however, that in general infective peritonitis operators must be prepared to lose many patients even when operation was undertaken early; while in the later stages, when there was distension and the pulse was becoming very rapid, the percentage saved would be very small.

